# Assessing the effect of PrLD proteins on Tau‐induced neurodegeneration

**DOI:** 10.1002/alz70855_107652

**Published:** 2025-12-25

**Authors:** Isabella Rose Juan, Isabella Aguirre‐Lamus, Adulfo Anaya Amador, Khondker Salim, Cassidy Dang, Dylan Lawrence Timperman, Joana Wei, Eon Lim, Ismael Al‐Ramahi

**Affiliations:** ^1^ Baylor College of Medicine, Houston, TX, USA; ^2^ Center for Alzheimer's and Neurodegenerative Diseases ‐ BCM, Houston, TX, USA; ^3^ Jan and Dan Duncan Neurological Research Institute, Texas Children's Hospital, Houston, TX, USA

## Abstract

**Background:**

A component of Alzheimer's Disease (AD) pathology centers on hyperphosphorylation and aggregation of tau into neurofibrillary tangles (NFTs). Because the catalyst and mechanism of pathological tau aggregation have yet to be precisely defined, it is important to consider the network in which tau may operate. AD is one of many proteinopathies, established by aggregation and misfolding of key proteins. Though not a feature of tau, other proteinopathy‐related proteins contain prion‐like domains (PrLDs), named for compositional similarity to yeast prion domains. PrLD proteins accelerate toxicity due to their aggregation proneness and promotion. The spread of tau during the progression of AD has been described as seed‐like in general. Additionally, tau is intrinsically disordered, much like PrLDs. These similarities suggest an environment in which the aggregation qualities of PrLD proteins may exert their own effects as seeds or even act as scaffolds in the context of tau accumulation. This study aims to uncover the functional relationship between tau and PrLD proteins *in vivo* using a *Drosophila* tauopathy model.

**Method:**

Using the PLAAC PrD detection algorithm we identified 90 proteins predicted to be PrLDs both in *Drosophila* and humans. Next, we screened the fruit fly homologs using loss of function and overexpression strains for genetic modifiers of the tau phenotype.

**Result:**

After extensive assessment of tau and PrLD interactions, we uncovered 36 alleles in which altered expression modified either external eye, retinal degeneration, or progressive motor performance phenotypes in our *Drosophila* tauopathy model. To further characterize the interaction between tau and PrLD dynamics, we assessed the effect of the hit PrLDs on tau accumulation in the context of both the *Drosophila* CNS and human RENcell neurons. Additionally, we characterized the interaction of tau and the hit PrLDs at the histological level in *Drosophila* and the mouse PS19‐P301S model.

**Conclusion:**

Ultimately, we identified a number of PrLDs whose knockdown ameliorates tau‐induced neurodegeneration, suggesting that they promote toxic tau accumulation. This is supported by colocalization of phosphorylated tau and PrLD proteins. Furthermore, tau protein levels are decreased by modifying PrLD expression, making these PrLD proteins attractive therapeutic targets in the context of AD.

